# Investigating the Feasibility and Safety of Osseointegration With Neural Interfaces for Advanced Prosthetic Control

**DOI:** 10.7759/cureus.82567

**Published:** 2025-04-19

**Authors:** Emma Shiwen Leung, Mohammad Mofatteh

**Affiliations:** 1 Neurosurgery, Neuro International Collaboration (NIC), London, GBR; 2 School of Medicine, Dentistry and Biomedical Sciences, Queen's University Belfast, Belfast, GBR

**Keywords:** advanced prosthetics, brain computer interface, neural interfaces, neurosurgery, osseointegration, peripheral nerve interfaces, prosthetic control, regenerative peripheral nerve interfaces

## Abstract

Osseointegrated neural interfaces (ONI), particularly in conjunction with peripheral nerve interfaces (PNIs), have emerged as a promising advancement for intuitive neuroprosthetics. PNIs can decode neural signals and allow precise prosthetic movement control and bidirectional communication for haptic feedback, while osseointegration can address limitations of traditional socket-based prosthetics, such as poor stability, limited dexterity, and lack of sensory feedback.

This review explores advancements in ONIs, including screw-fit and press-fit systems and their integration with PNIs for bidirectional communication. ONIs integrated with PNIs (OIPNIs) have shown improvements in signal fidelity, motor control, and sensory feedback compared to popular surface electromyography (sEMG) systems. Additionally, emerging technologies such as hybrid electrode designs (e.g., cuff and sieve electrode (CASE)) and regenerative peripheral nerve interfaces (RPNIs) show potential for enhancing selectivity and reducing complications such as micromotion and scarring. Despite these advances, challenges remain, including infection risk, electrode degradation, and variability in long-term signal stability.

Osseointegration combined with advanced neural interfaces represents a transformative approach to prosthetic control, offering more natural and intuitive movement with sensory feedback. Further research is needed to address long-term biocompatibility, reduce surgical invasiveness, and explore emerging technologies such as machine learning for personalized ONI designs. The findings of this review underscore the potential of ONIs to enhance embodiment and quality of life for amputees and highlight current pitfalls and possible areas of improvement and future research.

## Introduction and background

It has been estimated that approximately 57.7 million people globally are living with limb amputations caused by trauma. Major contributors to these amputations include falls (36.2%), road traffic accidents (15.7%), other transportation-related injuries (11.2%), and mechanical forces (10.4%) [1]. In the United States alone, 1.9 million people are currently living with limb loss, with this number expected to double by 2050 due to multiple factors, including the growing incidence of amputations as a complication of diabetes mellitus [2]. This rising global burden of limb loss highlights the increasing importance of advanced prosthetics, which are a promising future direction in restoring mobility and improving the quality of life for patients. 

From a study in 2023, the leading cause of upper limb amputation was trauma, followed by vascular complications of disease [3]. The evolution of prosthetic limb technology has transformed the lives of millions of amputees worldwide. However, conventional prosthetics often fail to provide intuitive control or meaningful sensory feedback, leaving users with limited functionality and connection to their artificial limbs [4]. With the advancement of biomedical engineering, the integration of neural interfaces with prosthetic devices has emerged as a promising solution to address these limitations, aiming to bridge the gap between the nervous system and prosthetics, allowing users to control artificial limbs intuitively using neural signals and potentially regain sensory perception. 

Current prosthetic devices have several limitations, including limited durability, unstable electromyography (EMG) readings, the necessity for precise fitting, and a lack of sensory feedback. Surface electrodes, which are separated from muscles by layers of soft tissue, fat, and skin, are prone to signal degradation due to crosstalk from adjacent muscles, resulting in unclear signals for the user [5]. Users often form a new prosthesis category separate from their hands in their parietal cortex, even when the prosthetic visually resembles a hand, showing that neural visual embodiment does not predict successful adoption of wearable technology by the human brain [6]. Therefore, prosthetic control based on neural interfaces offers significant advantages, including enhanced dexterity, more fluid movement, and the possibility of feedback systems that restore a sense of touch, pressure, and temperature, greatly increasing the embodiment and thus quality of life for prosthetic patients. This requires a connection of the nervous system to the prosthetic limb, using methods such as osseointegration paired with peripheral nerve interfaces (PNIs) to capture and decode neural signals to generate precise motor commands while incorporating sensory feedback mechanisms, allowing the prosthetic to feel more natural and controllable for the patient [7]. Osseointegrated neural interfaces (ONIs) are currently in early clinical stages, with most studies involving small cohorts or case reports. Large-scale human trials are still limited, and this gap should be addressed in future studies.

This review article aims to investigate the current and emerging methods for connecting prosthetic limbs with neural interfaces, examining advancements in osseointegration and PNIs, with a particular focus on how these technologies enable intuitive control of prosthetics and the mechanism of sensory feedback used in each method. The review combines descriptive and critical elements. In the Discussion section, we included a more explicit appraisal of technological limitations, such as long-term signal stability and infection risks, while maintaining a comprehensive overview of advancements.

## Review

Methods

Literature Search Strategy

A systematic literature search was conducted to identify studies investigating osseointegration (OI) combined with neural interfaces for advanced prosthetic control. The databases searched included PubMed, Institute of Electrical and Electronics Engineers (IEEE) Xplore, Scopus, and Google Scholar. Search terms included combinations of keywords such as "osseointegration," "neural interfaces," "peripheral nerve interfaces (PNIs)," "regenerative peripheral nerve interfaces (RPNIs)," and "prosthetic control." The search was restricted to English-language publications from 2010 to 2025 to ensure relevance to current technological developments.

The following Boolean search string was used: ("osseointegration" OR "bone-anchored prosthesis" OR "direct skeletal attachment") AND ("neural interface" OR "brain-machine interface" OR "BMI" OR "brain-computer interface*" OR "BCI" OR "peripheral nerve interface*" OR "implantable electrode*")**

Inclusion and Exclusion Criteria

Studies were included if they focused on the integration of OI with neural interfaces (e.g., PNIs, RPNIs) for prosthetic control; reported clinical outcomes, technological innovations, or experimental findings; or involved either human or animal models.

Studies not directly addressing prosthetic control or neural interfaces, articles lacking empirical data or a detailed methodology, and duplicate publications or review articles without original data were excluded.

Selection Process

A total of 407 articles were initially identified. After title and abstract screening, 298 were excluded for not meeting the inclusion criteria. The remaining 109 full-text articles were reviewed in detail, of which 49 were excluded due to insufficient data or off-topic focus. Ultimately, 60 articles were included in the final analysis.

Data Extraction and Synthesis

The following data were extracted from each study: study design (e.g., clinical trial, preclinical animal study); type of neural interface used (e.g., cuff electrodes, sieve electrodes, RPNIs); reported outcomes (e.g., signal stability, sensory feedback, infection rates); limitations and proposed future directions

Statistical Analysis

Due to heterogeneity in study designs and reported outcomes, a meta-analysis was not performed. Instead, descriptive statistics were used to summarize findings, including reported success rates, complication rates, and performance metrics (e.g., signal-to-noise ratios (SNRs)).

Discussion

Mechanisms of Osseointegrated Neural Interfaces

**Figure 1:**
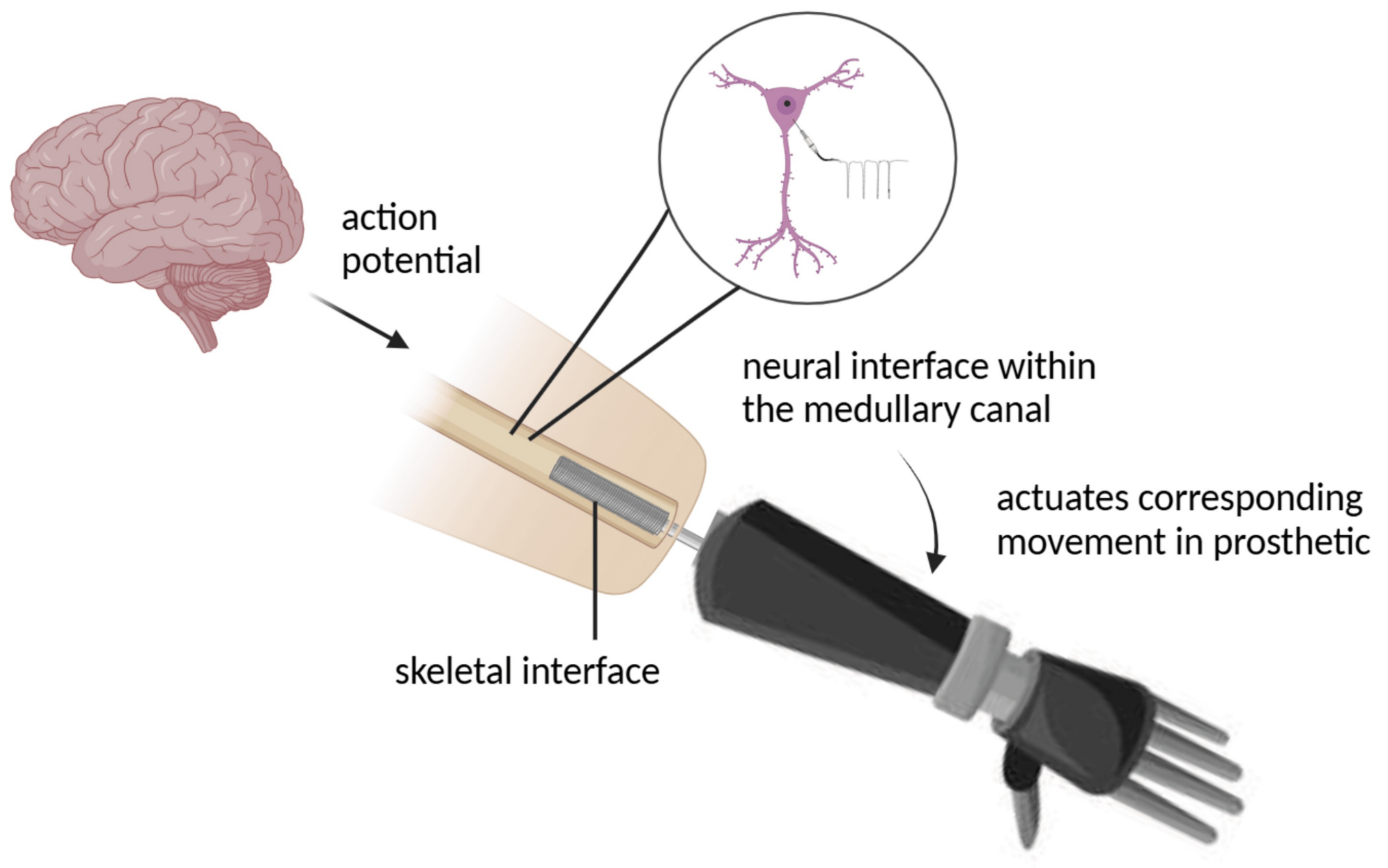
A summary of the osseointegrated neural interface mechanism. An action potential is sent from the brain to the peripheral nerve, which is read by the electrodes surrounding the nerve. These electrodes capture the electrical signals and transmit them to a decoder, typically an algorithm or computational model trained to interpret neural signals. The decoder translates the neural activity into specific motor commands, which are then used to actuate the intended movement in the prosthetic limb. The authors created the figure using BioRender (BioRender, Toronto, Canada).

OI refers to a surgical procedure that aims to create a permanent connection between the bone tissue and metal implants [8]. A major limitation in prostheses is the lack of long-term stability due to microdamage caused by compliance mismatch as well as the continuous movement of soft tissue [9]. In addition, the interface between the skin and bone is often compromised in traditional press-fit socket systems by subcutaneous fat, which increases the risk of chronic wound infections [10]. This issue has been addressed by developing an ONI for prosthetic control, which integrates peripheral nerves into the medullary canal of long bones within a bidirectional closed-loop system. Such integration systems enable both motor and sensory feedback within the prosthetic, fostering synergistic control and enhancing the user’s sense of embodiment [11]. ONI, which are permanent percutaneous and permucosal devices, offer a solution to the long-term stability challenges associated with traditional socket prosthetics while also improving the sense of embodiment. The development of an ONI involves establishing a nerve interface connected to an advanced prosthetic. Currently, the most used nerve interfaces are myoelectric control, brain-computer interfaces (BCIs), and PNIs (Figure 1).

Percutaneous osseointegrated interfaces provide a stable and continuous bidirectional communication channel using implanted electrodes in the peripheral nerves and muscles [5]. The neural interface components and electrodes are placed within the intramedullary canal, which supports nerve regeneration and maintains physiological activity to improve the longevity and functional performance of the electrodes. Selective electrodes, such as sieve electrodes, are crucial due to their capacity to deliver high fidelity in neural signal recording. While these electrodes enable precise activation of individual neural fascicles, their invasiveness poses challenges, including an increased risk of inflammation and scar tissue formation, which may jeopardize the long-term stability of the interface. This location serves as a protective space for complex electrode configurations by reducing electrical crosstalk and nerve compression from adjacent muscles, improving extraneural electrodes’ spatial selectivity [11]. A pilot study demonstrated the feasibility of establishing a neural interface within the bone by transposing nerve terminals into the medullary canal of long bones. The bone structure provides the necessary stability for the transposed nerves, allowing them to remain physiologically active. Additionally, electrical stimulation of the residual nerve within the bone successfully elicited compound nerve action potentials at 12 weeks post-transposition [12], indicating that nerves can be transposed into bones effectively for ONI systems. 

Types of Osseointegration

**Figure 2:**
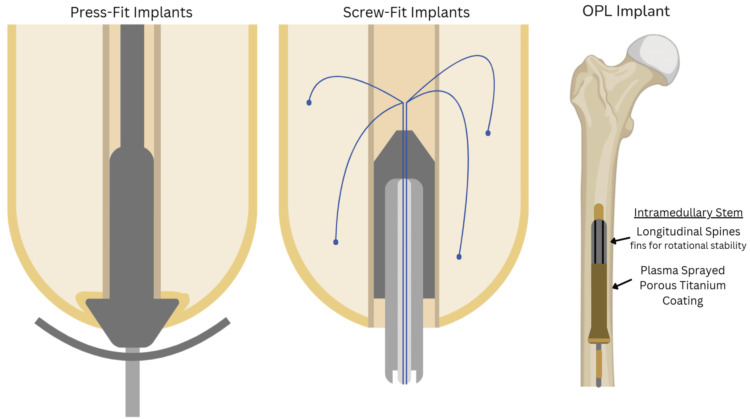
Press-fit systems, screw-fit systems, and osseointegrated prosthetic limb (OPL) systems. Screw-fit systems thin out the subcutaneous fat tissue layer covering the periosteum to create a tight skin-bone interface, which prevents chronic infection [10]. Screw-fit implants also allow electrodes to exit the abutment. On the far right is an OPL implant within the femur with an intramedullary stem and plasma-sprayed porous titanium coating. There are longitudinal spines on the intramedullary stem for rotational stability [13]. The authors created the figure using BioRender (BioRender, Toronto, Canada). OPL: osseointegrated prosthetic limb

There are two primary types of OI implants: the cannulated screw-fixation implant, which relies on bone on-growth rather than in-growth to improve its long-term viability; and the press-fit macroporous surface structure implant, which encourages bone in-growth and penetration, offering better prospects for long-term integration (Figure 2) [10,13].

Screw-fit systems provide more stable and durable fixation than press-fit systems. By thinning or replacing the skin near the periosteum with a split-thickness skin graft, screw-fit systems ensure strong adhesion to the bone while minimizing granulation tissue formation and thereby lowering the risk of infection. In contrast, press-fit systems rely on the bone’s natural press-fit, which is more susceptible to complications arising from the thick subcutaneous fat layer at the skin-bone interface. Such conditions can increase the risk of chronic wound infections and compromise the long-term stability of the prosthetic attachment [10]. 

The Osseointegrated Prostheses for the Rehabilitation of Amputees (OPRA) system, originally reported by Ortiz-Catalan [5], has provided the foundation for many screw-fixation OI systems in use. The modular OPRA implant system uses a two-stage surgical procedure where a titanium screw-like fixture is implanted within the intramedullary cavity of the bone at the stump to allow for OI through bone-on-growth. An abutment extends percutaneously from the fixture, serving as the anchor for the prosthetic limb. The mechanical connection between the fixture and abutment, secured by an abutment screw, ensures stable load transfer. 

Even though ONIs provide high stability, the integration of an osseointegrated prosthetic is extremely time-consuming due to the highly invasive nature of the prosthetic connection. In an ONI setup, as nerves must be connected to electrodes within the bone with sufficient length and medullary space, the surgical integration of the ONI requires careful planning to ensure optimal nerve placement and functionality [14]. Surgeons must preserve sufficient nerve length beyond the residual stump to transpose the nerves into the medullary canal without inducing tension [11]. However, this is not always feasible due to the mechanism of injury as well as surgical complications. 

Quantifying the available medullary cavity volume and diameter at different amputation levels through computerized tomography (CT) imaging and virtual implantation is crucial in designing ONIs. This process determines how electrodes can be incorporated, as the available space dictates the size and configuration of the interface. For instance, a large-diameter stem design requires more bone removal than smaller-diameter stem designs. The design and placement of electrodes within the ONI should account for the variability in nerve morphology, such as changes in nerve circumference along its length, to optimize the SNR and minimize interference from surrounding tissues [11], emphasizing the importance of a personalized approach for each setup. To enhance precision and efficiency in designing ONIs, further research should focus on completely automating the design process. In the future, automated machine learning algorithms could analyze CT imaging data to quantify the medullary cavity volume and diameter, generating detailed 3D models of the residual limb based on patient-specific data to predict the most suitable configurations based on patient-specific data [15]. Automation of this process may reduce the turnaround time for ONI design and possibly minimize human error. It may also be possible to use these models to simulate virtual implantations to ensure optimal fit and alignment, further improving the patient experience [15]. 

The procedure starts with the implantation of a titanium fixture into the intramedullary canal, which is then left undisturbed for several months to allow OI. Then, a percutaneous component is attached to the skeletal structure of the prosthetic limb. The neuromuscular structures within the residual limb, including native muscles and free muscle grafts, are restructured and fitted with implanted electrodes. In upper limb setups, epimysial electrodes are positioned on each head of the biceps muscle and on the lateral and long heads of the triceps muscle, while cuff electrodes with a mixed tripolar contact configuration are wrapped around fascicles of the median nerve [16]. 

In practice, electrode placement in ONIs and PNIs is determined through a combination of surgical planning, imaging, and patient-specific considerations. Considerations include surgical planning and imaging, where preoperative imaging (e.g., CT or MRI) is used to assess the medullary canal's volume and nerve morphology, ensuring adequate space for electrode placement. Virtual implantation simulations may guide the design of personalized electrode configurations. Larger nerves (e.g., median or ulnar) are preferred for interfacing due to their fascicular organization, allowing selective dissection without disrupting nerve function. The number of reinnervation sites depends on fascicle availability and surgical feasibility. Cuff electrodes wrapped around nerves extraneurally; placement avoids excessive tension to prevent micromotion. In contrast, sieve electrodes require nerve transection and regeneration through perforations and are positioned at nerve terminals within the medullary canal for stability. Hybrid designs combine cuff and sieve features to balance selectivity and invasiveness. Intraoperative testing includes electrical stimulation during surgery, which verifies optimal electrode placement by eliciting target muscle responses or sensory feedback. SNR is assessed to ensure clear signal acquisition. Postoperative calibration can use machine learning algorithms to decode neural signals post-implantation, refining electrode utility for prosthetic control. Long-term stability is monitored to address issues like scar tissue or signal degradation [16].

At its most basic design, this system integrates with a PNI, which includes one spiral cuff electrode, two bipolar electrodes, and four monopolar epimysial electrodes. Notably, the bipolar epimysial electrodes used in this system mitigate myoelectric crosstalk, enhancing the SNR. This improvement results in either a reduction of noise, an increase in the effective signal amplitude, or both, enabling superior signal clarity. In addition, the modular design of the system enables further upgrades to more selective neural interfaces, such as intrafascicular and flat nerve interface electrodes [5]. This is important because studies using intrafascicular electrodes suggest that motor nerve fibers may remain viable in long-term amputees, despite cortical reorganization and axotomy-induced degeneration, implying that directly interfacing with peripheral nerves in OI could still detect motor neural activity, even in cases of myelinated motor fiber degeneration. However, it is worth emphasizing that this system was only tested on one patient, which limits the generalizability of the results [5]. Further studies are required to validate and expand these findings. 

Additionally, combining OI with advanced surgical techniques, such as targeted muscle reinnervation or regenerative peripheral nerve therapy, can provide independent control of myoelectric sites. These techniques could further expand the functional capabilities of the prosthesis [5].

Tactile perception was also successfully reproduced over the long term through direct electrical stimulation of peripheral nerves, even after long-term amputation. Combining this method with surgical techniques such as targeted muscle reinnervation or RPNIs could potentially provide additional myoelectric control sites, enabling reliable and independent control of multiple degrees of freedom [17]. Similarly, press-fit OI implants involve the direct insertion of a porous titanium implant into the bone. Over time, the bone grows into the porous surface of the implant, creating a secure and permanent bond [18]. 

**Figure 3:**
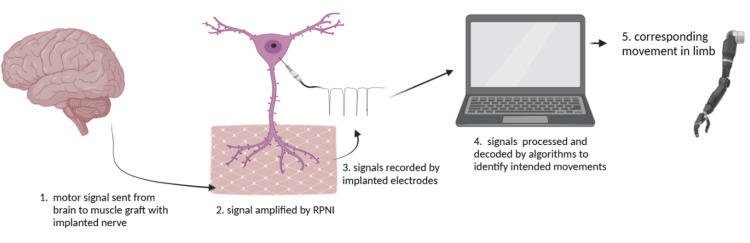
An overview of the regenerative peripheral nerve interface (RPNI) mechanism. First, a motor signal is sent from the brain to the muscle graft containing the implanted nerve. Then, the signal is amplified by RPNI and recorded by implanted electrodes. Signals are processed and decoded by algorithms to identify intended movements. Finally, the corresponding movement is actuated in the limb. The authors created the figure using BioRender (BioRender, Toronto, Canada).

The osseointegrated prosthetic limb (OPL) implant is a notable press-fit implant, shown in Figure 3, which includes an intramedullary stem component designed for initial stability and sustained anchorage through bone ingrowth. The implant's proximal end has smooth surfaces with sharp splines to enhance rotational stability, while the distal end is coated with microporous particles to provide axial stability and promote bone ingrowth. Such features enhance long-term OI by progressively strengthening the connection between bone and implant [13]. 

Similarly, the skin and bone integrated pylon (SBIP), as seen in Figure 2, is a type of press-fit OI system designed to enhance the long-term attachment of prosthetic limbs to bone and skin. Utilizing porous titanium, the SBIP promotes bone ingrowth into its porous structure, providing screw-free mechanical stability. It also encourages soft tissue integration, helping to seal the skin around the implant, which is crucial for preventing infection while also improving osseoperception [18]. However, data on human subjects are lacking, as reported results were from animal studies (16 cats and four pigs). 

Advantages of Osseointegration

OI has proven to be a relatively safe and effective method for reconstructing and rehabilitating lower limb amputees, showing marked improvements in post-operative quality of life and functional outcomes [13].

The osseointegrated nature of the prosthesis also ensures that its controllability is unaffected by limb position, skin interfaces, environmental conditions, or electromagnetic interference. The system is also capable of predicting distal motions through myoelectric pattern recognition at the stump level. This allows the users to experience a more natural range of motion comfortably, contributing to a greater sense of embodiment, which is essential for prosthetic users. Importantly, no muscle fatigue was reported, and the time spent wearing the prosthesis increased by approximately six hours per day compared to traditional surface electromyography (sEMG), with a significant reduction in phantom limb pain by 40% [5].

In an extended home-use study involving a 63-year-old patient with an osseointegrated interface, continuous prosthesis usage increased, and functional metrics showed up to 26% improvement in control assessments and 76% in perceived workload evaluations. The study also noted enhanced user integration, characterized by increased torque loading and improved control performance. The required EMG signal magnitude for prosthesis control decreased subsequently, but performance remained stable, indicating improved control efficiency. This was achieved by a machine learning-based myoelectric pattern recognition algorithm [19]. Future larger-scale studies are required to validate and extend these findings. 

Compared to other interfacing methods, OI is linked to a reduced rate of lower extremity fractures and lower incidences of seroma formation, cutaneous infections, skin reactions, and avascular skin flap necrosis. While percutaneous wires for signal transmission present a high risk of cutaneous infection, OI mitigates this by incorporating an enclosed communication channel that protects the signal transmission pathway from external contaminants [10].

A ten-year study of 51 patients with OPRA demonstrated significant enhancements in prosthetic use, mobility, and overall quality of life as measured by the Questionnaire for Persons with a Transfemoral Amputation (Q-TFA) scores [20]. Revision-free survival rates of 83% for implant revisions, 65% for deep infections, and 17% for mechanical complications were observed. Notably, mechanical complications, which were considered the most severe adverse events, became more common in the latter five years of the study, which is concerning for the long-term reliability of the implants, highlighting the need for advancements such as SBIP to ensure effective long-term usage of osseointegrated prosthesis [20]. Similar findings were reported in other studies [13].

It is important to note that no studies to date have reported additional amputation or fatalities associated with OI implants [21], indicating its safety and feasibility. However, due to the highly invasive nature of osseointegrated prosthetics, infections are extremely common. 

Alternatively, the design of percutaneous porous implants such as SBIP also includes key features such as optimized porosity, pore size, volume fraction (VF), and particle size to enhance tissue integration. Theoretically, a VF greater than 50% can support cellular ingrowth, which promotes a more secure integration of both bone and soft tissue [18]. Even though the SBIP can initially integrate well with the skin, skin epithelial downgrowth along the porous surface can disrupt the skin integrity in the long term, increasing the risk of infection. Studies show that this breakdown can occur within 24 months, potentially leading to implant failure and infection if the skin-implant seal is not maintained.

In the proposed infection-resistant SBIP prosthetic, only one of the six animals (16.67%) developed an infection, which is significantly lower than the other studies mentioned above. In this animal study, six cats were implanted for 28 to 70 days and fitted with a unilateral passive transtibial prosthesis for 61 to 122 days to assess the performance of SBIPs under natural loading conditions. This result is promising for the future development of SBIPs, which outperformed the OPRAs in terms of infection [18].

Although no studies on the OPRA system specifically report periprosthetic fractures with loosening, femoral neck fractures are reported at a rate of 2% to 11%, higher than in the general population [22]. The femoral neck and the area near the OPRA implant are subjected to weight-bearing and rotational stress, which makes femoral neck fractures a comparison index for evaluating the prognosis of OPRA implants. Also, patients with OI implants can experience femoral neck fractures at a higher rate than the general population due to unbalanced weight distribution, thereby emphasizing long-term outcome assessment [21]. Future studies are required to investigate sex-specific and age-associated outcomes of femoral neck fractures, as such variables can influence the findings. 

Emerging electrochemical methods, such as metallic implants, can circumvent these limitations by delivering localized electrical stimulation to prevent or treat bacterial biofilms at the implant site, addressing a major cause of implant-associated infections. Additionally, direct current (DC) electrical stimulation could monitor implant performance and deliver electrical stimuli when necessary. This technique utilizes electrochemical impedance spectroscopy (EIS) to measure voltage or current responses for the detection of changes in impedance, which provides real-time data on the implant's OI status. A decrease in impedance could signal poor integration or loosening, while abnormal readings might indicate infection. This non-invasive approach offers a promising way for clinicians to monitor OI and infection risks, potentially reducing complications associated with OI implants [23]. 

Despite all advances, osseointegrated interfaces serve primarily as a method of physical connection between the patient and the prosthetic, rather than offering intuitive control. ONIs, however, combine OI with control mechanisms such as myoelectric systems, PNIs, or BCIs to enable intuitive control. Importantly, ONIs do not inherently provide sensory feedback. While touch sensors may be incorporated into prosthetics using OI, their primary function aligns more closely with the control and feedback systems in PNIs, which will be discussed next. 

Comparison of ONIs and Traditional Surface EMG Systems in Clinical Outcomes

Signal fidelity and control precision: ONIs provide higher signal fidelity due to direct neural interfacing, enabling precise motor control and bidirectional communication (sensory feedback), and sEMG is susceptible to noise, crosstalk, and signal degradation from soft tissue layers, limiting control accuracy.

Functional performance: ONI users achieve more natural and intuitive movements, with studies reporting improved dexterity (e.g., 26% better control assessments) and reduced reliance on compensatory motions, whereas sEMG is limited to gross movements and struggles with complex tasks due to lower signal resolution.

Sensory feedback: ONIs enable bidirectional haptic feedback (e.g., touch, pressure), enhancing embodiment and reducing phantom limb pain by 40%, whereas sEMG typically lacks sensory feedback, relying on visual or auditory cues.

Long-term stability: ONIs offer durable integration (e.g., 83% implant survival at 10 years) but face challenges like infection (e.g., 34% superficial infection rates) and electrode degradation, while sEMGs are non-invasive but require frequent recalibration and socket adjustments due to skin interface issues (e.g., sweating, fit changes).

User experience: ONIs increase daily prosthesis use by ~6 hours compared to sEMG, with higher comfort and reduced muscle fatigue, while sEMG is often associated with discomfort, socket irritation, and shorter usage periods.

Peripheral Nerve Interfaces

PNIs record and modulate neural activity in peripheral nerves through electrodes, which can be translated into actuator movement in prosthetics. Implantable PNIs allow more selectivity in recording and stimulation compared to non-invasive methods but face long-term challenges due to micromotion and signal degradation [24]. Electrode stimulation is most used in prosthetics, including monopolar, bipolar, cuff, and sieve electrodes to allow bidirectional communication between the prosthetic and the patient [25]. Combined with OI, which can house these electrodes in the medullary canal, intimate neural interfacing can be achieved. Direct percutaneous connectivity and maximizing contact area between the endosteum and the OI region [11] enhance access to the organized structure of axons in peripheral nerves. This design improves signal specificity by reducing signal overlap, increasing selectivity, and lowering stimulation thresholds [12]. As a result, users can perform a wider range of tasks with greater ease and control, leading to a more intuitive and responsive experience. 

Most importantly, the structural and physiological function of the transposed nerve is maintained in PNI. Over time, compound nerve action potentials improve, and a greater number of channels can generate somatosensory evoked potentials [11].

Balancing the usage of monopolar or bipolar electrode configurations is important for the quality of the signals. Monopolar electrodes are typically preferred when the implant system has limited electrode contacts, as they allow for the instrumentation of more muscle targets. However, this results in a lower SNR compared to bipolar electrodes [26]. Bipolar configurations, in contrast, offer a higher SNR for precise prosthetic control. The choice of electrode configuration depends on the patient-specific characteristics, including the presence of nerve fascicles and the balance required between signal clarity and the number of muscles targeted for reinnervation [27].

The total number of reinnervation sites is determined by the number of fascicles that can be dissected without disrupting the natural crossings of fascicles within the nerve trunk [17,28]. Larger diameter nerves provide more opportunities for fascicle dissection. Electrode selection also depends on the type of muscle target [17,28]. For example, intramuscular electrodes are preferred for nonvascularized free muscle grafts because epimysial electrodes could impair blood diffusion across a large surface area. In the upper limbs, the ulnar and median nerves showed reinnervation at around five months post-surgery, while the radial nerve showed similar reinnervation at 11 months, leading to distinct myoelectric signals across different electrodes and a significant increase in SNR [29]. Zbinden et al. successfully recorded five degrees of freedom (DOFs), the independent coordinates required to fully describe the position or movement of a body or system, 48 weeks post-implantation using this method [16].

The two main types of electrodes used in ONI are cuff and sieve electrodes (CASE). We will discuss the mechanisms, advantages, and disadvantages of these electrodes within a PNI below. 

Cuff Electrodes

Cuff electrodes usually consist of a cylindrical silicone tube that encircles the nerve [30] in which platinum-iridium (Pt/Ir) wires are concentrically threaded. Due to their electrical conductivity and biocompatibility, these wires record and stimulate nerve activity [31]. After surgically positioning the terminal end of a severed nerve within the medullary canal of the bone, which protects the nerve from mechanical and electrical disturbances, a cuff electrode forms an interface with the nerve tissue, wherein the signals generated by the interfaced nerve fibers can be detected by the electrode wires in the cuff. The electrode wires are connected to printed circuit boards, which link to the osseointegrated prosthetic abutment, enabling real-time monitoring of nerve activity [12]. As a whole, this configuration provides a stable, percutaneous connection for the electrodes. Often, the entire assembly is encapsulated in dental acrylic to protect it from surrounding tissue. Both efferent and afferent compound nerve action potentials (CNAPs) can be recorded using cuff electrodes in ONIs [12].

Haptic feedback can be delivered through cuff electrodes by stimulating afferent nerve fibers to deliver sensations such as touch, pressure, and potentially proprioception. However, integrating haptic feedback into cuff electrodes is uncommon due to low selectivity; stimulating a broad range of nerve fibers often results in mixed or unnatural sensations [32]. Even with selective stimulation, decoding the complex sensory patterns needed for natural haptic perception is challenging. Tan et al. stimulated the proprioceptive sensation of finger flexion through a cuff electrode on the median nerve, proving that cuff electrodes can provide proprioceptive feedback [33]. Accurate replication of the intricate interactions between sensory receptors and neural pathways to process tactile and proprioceptive input via artificial stimulation requires immense developments in feature engineering. 

Currently, the focus is on improving cuff electrode selectivity and stimulation strategies while exploring the neural basis of haptic perception for feature engineering. For example, Tan et al. proposed that using cuff electrodes with multiple, spatially distributed contacts can enhance selectivity and allow for more precise control over the sensations elicited. In addition, better decoding models, such as musculoskeletal models, traditional machine learning models, deep learning models, and hybrid models, can be applied to the recorded nerve activity to isolate specific sensory signals, which can be replicated to deliver more accurate feedback [33].

Integrating cuff electrodes with sensors embedded in the prosthetic limbs for closed-loop feedback systems in the future may enable real-time adjustments to stimulation parameters based on the prosthetic’s interaction with the environment, possibly providing more natural and intuitive haptic feedback. 

Cuff electrodes are relatively non-invasive as they are designed to wrap around peripheral nerves without penetrating the epineurium. Thus, they are easier to implant, and the surgical procedure is less complex compared to more invasive interfaces such as sieve or interfascicular electrodes, leading to a lower risk of nerve damage and faster recovery times, providing a balance between recovery intensity and functional selectivity for prosthetic control [29]. Stable impedance values in the range of approximately 2.7-3.1 kΩ and consistent sensory thresholds over one to two years were reported, suggesting minimal tissue reaction and nerve trauma [33]. Such stability is consistent with faster recovery times post-implantation, as less nerve disruption typically correlates with more rapid functional recovery. Additionally, the low stimulation thresholds (<50 nC) recorded by Yildiz et al. (2020) support that the minimal invasiveness of cuff electrodes reduces the extent of nerve injury, which is also associated with improved recovery outcomes [29]. 

Chronic stability of the cuff and its independence from nerve regeneration through the electrode, such as sieve electrodes, causes less direct physical pressure on nerve fibers [34]. This minimizes the risk of micromovements and mechanical stress, which may cause signal instability or electrode failure over time [35]. In addition, the more flexible and biocompatible materials used in cuff electrodes can withstand the mechanical strain associated with limb movement to conform to the nerve shape, making cuff electrodes very adaptable. Such adaptability increases the long-term stability and functionality of cuff electrodes, as demonstrated by stable and selective stimulation over a 24-month period reported by Tan et al. [33]. They demonstrated that in two human amputees, multi-contact cuff electrodes maintained stable stimulation thresholds and impedance over extended periods, up to 24 months. In their study, 97% of the 35 active channels showed either no significant change or a decrease in threshold over time. Even though cuff electrodes do not provide the single-fiber selectivity seen in more invasive neural interfaces, they provide sufficient selectivity for daily use and general functionality [36]. In Tan et al. [33], 19 out of 20 active contacts elicited a sensory response that collectively spanned approximately 15 unique percept areas, while in another trial, all 16 contacts produced percepts over about 10 unique areas. These numbers illustrate that, while the interface is less selective than penetrating electrodes, it still affords distinct, reproducible sensory feedback. 

It is also possible to have a multi-contact configuration where cuff electrodes with multiple spatially distributed contacts target specific fascicles within the nerve, allowing selective activation of specific muscle groups. This results in more precise control of prosthetic movements, enhancing the overall patient experience. However, cuff electrodes may lead to inflammation, fibrous tissue formation, and scarring around the electrode [36].

However, integrating cuff electrodes into long-term PNIs is challenging due to their high contact with the nerve. The implantation of any foreign object triggers a response in the surrounding tissue, leading to inflammation, fibrous tissue formation, and scarring around the electrode. These complications will ultimately compromise the performance of the electrodes by increasing impedance between them and the nerve, which weakens and distorts the recorded signals [36]. In response to this change, the PNI will require higher electrical currents to achieve the same level of stimulation as before in the same PNI setup, which over time can lower the effectiveness of prosthetic control and sensory feedback while increasing the risk of tissue damage from excessive electrical stimulation [29]. In addition, the implantation process alone can cause mechanical trauma to the nerve fibers [12], which may disrupt nerve conduction and cause more long-term nerve damage. 

Since cuff electrode materials do not perfectly match the compliance of nerve tissue despite their flexibility, micromotion may happen between the electrodes and the nerve, especially during movement [12,35]. Micromotion causes microdamage to the cuff electrode contact and the nerve fibers, which may cause inflammation, scarring, and signal degradation, compromising the interface's long-term stability. Inflammation may cause fluctuations in the recorded signals, making it challenging for the computer inside the interface to extract features for the decoding model to translate into movement. Thus, prosthetic control will be less effective and reduce its reliability, increasing the patients' abandonment rate [12]. 

In addition, the material may also degrade over time, which may cause impedance, reduced signal quality, and eventual mechanical failure. This is caused by constant movement, temperature fluctuations, and exposure to biological fluids commonly experienced by the body. Also, changes within a nerve, such as growth, atrophy, or positional shifts within the interface, can compromise the stability of the PNI [29]. Thus, developing more biocompatible materials is needed to improve the long-term durability of cuff electrodes. 

The percutaneous wires connecting the cuff electrodes to external devices for signal processing and power are also prone to failure due to the constant movement and flexing of the wires, which causes damage to the insulation. The damage and possible breakage interrupt signal transmission and expose the surrounding tissue to electrical signals, increasing the risk of tissue damage and infection [37].

Cuff electrodes may also stimulate or record from a more extensive range of nerve fibers than intended due to their lower selectivity. Not only does this limit the precision of control and the quality of sensory feedback [32,38], but a cuff electrode designed to stimulate a specific nerve may also activate nearby sensory nerves, leading to unintended sensations. Inversely, a cuff electrode designed to record signals from one nerve may also capture activity from nearby nerve fascicles. This makes it harder for the computer's feature engineering and decoding models to isolate and recognize signals for the intended movement [35]. Currently, strategies such as using multi-contact cuffs with spatially distributed electrodes and using artificial intelligence instead of traditional machine learning for decoding models to isolate signals are being explored to improve the selectivity of cuff electrodes and improve sensation and control [31].

Despite such advances, cuff electrodes are limited in recording distinct efferent motor action potentials due to the small signal amplitude. Sieve electrodes, another common interface used in ONIs, overcome this problem at the expense of a more difficult recovery.

Sieve Electrodes

A sieve electrode is a flat, perforated disc containing multiple electrode contacts, allowing regenerating nerve fibers, which are surgically transected, to grow through the holes, integrating the device into the nerve structure [31]. This intimate interface provides high selectivity [32]. Sieve electrodes are usually made from biocompatible, flexible, electrically conductive materials resistant to body degradation, such as platinum/iridium, polyimide, and Parylene-C [29].

Sieve electrodes are a highly selective type of PNI due to their close contact with individual nerve fibers. In a proof-of-concept study, the addition of a sieve interface to a cuff electrode within the medullary canal enhanced the recording of efferent and afferent CNAPs across more channels. This enhancement is due to the sieve electrodes' ability to reduce the resistance of the epineurium by being in closer proximity to the nerve fibers, resulting in more effective stimulation with lower electrical input and stronger evoked responses [31].

Conducted in a rabbit amputation model, the study reported that sieve electrodes produced somatosensory evoked potentials (SSEPs) with higher peak amplitudes than cuff electrodes. Additionally, positioning sieve electrodes at the terminal end of the amputated nerve improved neural engagement over time compared to the cuff electrodes used in the same animal. However, this approach requires invasive nerve transection and depends on successful neural regeneration through electrode transit zones to integrate the PNI into the nerve [31].

Coker et al. developed the micro-channel sieve electrode to improve the interaction between the nerve fibers and the electrode. The micro-channel version has smaller perforations arranged in a grid pattern for nerve fiber regeneration to guide smaller groups of axons to achieve more intimate contact [32]. The fibers contact the electrode as the nerve regenerates through the micro-channels. This allows even more selective recording and stimulation of nerve activity with higher signal quality, improving both sensation and control of the prosthetic by targeting individual axons with high specificity to improve translation into prosthetic movement. Since the micro-channel design also acts as a spatial filter that concentrates the signals from the nerve fibers, it increases the SNR, creating a focused signal. Instead of the 1/r decay usually observed in a homogenous volume conductor, the potential within the microchannel decays linearly toward the edges, which means that the signals are more consistent and reliable regardless of the axons’ position in the channel. Such a design reduces variability in signal amplitude because of position, and less electrical current is needed to stimulate the nerve, which reduces the risk of damage to the surrounding tissue, as mentioned with cuff electrodes. In addition, the micro-channel sieve electrodes are made from biocompatible materials such as Pt/Ir and polyimide, making them suitable for long-term interfacing and reducing the risk of micromovement. 

Even without modifications reported by Coker et al., sieve electrodes have enhanced selectivity compared to other electrode types. Unlike extraneural electrodes, such as cuff electrodes, sieve electrodes are designed to interface directly with individual nerve fascicles [29]. High selectivity allows for finer motor control, resulting in more dexterous and natural movements, unlike cuff electrodes, which only allow general muscle activation [31]. High selectivity also allows sensations to be more targeted, which allows patients to perceive tactile sensations with greater resolution and spatial accuracy, improving embodiment and control [29].

The high invasiveness of sieve electrodes is associated with a greater risk of tissue damage and immune reactions. This may eventually cause scar tissue formation around the electrode, impeding signal transmission by reducing direct contact between the electrode and the nerve fiber, resulting in signal degradation over time [12]. To combat this problem, coatings that release anti-inflammatory drugs or bioactive molecules can be applied to minimize the immune response and scar tissue formation [29]. Also, smaller, more conformable electrodes could help reduce implant size and invasiveness, mitigating the foreign body response (FBR) to the electrodes [39].

Quinn and colleagues [39] point out that wired connectors are a significant failure point in implantable myoelectric sensors (IMES), especially with repeated movement due to the vulnerability of percutaneous connections. Sieve electrodes often rely on percutaneous leads, which may be prone to similar failure modes and create potential entry points for infection. Although this concern is not specific to sieve electrodes, Brånemark [40] discusses infection rates associated with osseointegrated prostheses in a four-year follow-up wherein there was a 34% risk of superficial tissue infection. This highlights the inherent infection risk in any implantable device with percutaneous connections, but it is also key to note that there were no peri-implant deep infections reported in this study, which is promising news for the wider adoption of osseointegrated PNI prosthetics in the future. 

Although the issue of electrode degradation in sieve electrodes is not directly addressed in ONI studies, Ehrensberger et al. [23] highlighted the importance of using appropriate electrical stimulation parameters and maintaining stable reference electrodes to control electrochemical processes at the electrode-tissue interface. Though focused on OI, these considerations emphasize the necessity to understand electrochemical interactions between implanted electrodes and biological tissues for long-term stability in the future. Table 1 provides a comparison between CASE.

**Table 1:** A comparison between cuff and sieve electrodes. nC: nanocoulombs; CNAPs: compound nerve action potentials; SSEP: somatosensory evoked potentials; EMG: electromyography

Feature	Cuff electrodes	Sieve electrodes
Invasiveness and Structural Design	• Non-penetrating, wraps around nerve without direct fiber contact, reduces surgical complexity and risk of damaging the nerve during implantation [12] • Less invasive, low threshold stimulation <50 nC required [29]	• Invasive design, placing a perforated disc within severed nerve ends, then nerve regeneration through the holes [31] • Direct contact with regenerating axons [29] • Increased risk of chronic inflammation and scar tissue; superficial infections occurred 41 times in 28 patients (54.9% infection rate over two years) [40]
Selectivity	• Lower selectivity, interacts externally with the nerve, targeting multiple fascicles [34] • Basic control for gross movements but may lack fine motor precision [38]	• Designed for intraneural placement, high selectivity by engaging individual nerve fibers or fascicles [41] • Finer control over movements, ideal for dexterous motor control in advanced prosthetics [31] • CNAPs improved between weeks three to five; somatosensory responses measurable by 12 weeks, indicating enhanced precise recruitment [31]
Signal Quality and Stability	• Table stimulation thresholds and impedance maintained up to 24 months [33] – 97% of 35 active channels: unchanged or decreased thresholds – Mean channel impedance: 2.7–3.1 kΩ • Low signal degradation: non-penetrating design minimizes tissue reaction and micromotion effects [35]	• High signal quality: 9 mm × 90 µm micro-channel design yielded peak amplitudes of 600–800 µV [32] • Enhances signal-to-noise ratio and minimizes variability from axonal placement [32] • Electrical potential decays linearly toward micro-channel edges, reducing fluctuations [32], leads to higher SSEP peak amplitudes [31] • Long-term stability can be compromised by tissue reactions [12]
Applications in Prosthetic Control and Sensory Feedback	• Can be used to deliver sensory feedback by stimulating afferent fibers [33] • Quality of haptic feedback less refined compared to more invasive methods [​32] • Reproducible sensory feedback: – Subject 1: 19/20 contacts yielded ~15 distinct percept areas – Subject 2: 16/16 contacts yielded ~10 distinct percept areas [33]	• Fine motor control, enabling dexterous movements [31] • Bidirectional communication potential [25] • EMG peak amplitudes ~10× higher with micro-channel sieve electrodes versus transverse intrafasicular multichannel electrode (TIME) electrodes, indicating broader, less localized activation, resulting in coarser haptic feedback [32]
Long-Term Viability	• Remain functional for up to 11 years [29] • Tissue scarring may impede performance over time [29] • Cause less nerve damage and maintain stability long-term [35]	• Common foreign body response, including chronic inflammation and scarring, may reduce signal quality [12] • Stable pattern recognition and improved motor control in one patient over one year [5] • Achieved selective muscle recruitment over six months [42] • Cases of constrictive axonopathy observed in some animals after six to 12 months [43] and reported disorganization in dorsal column nuclei after 30 months [44]

Future Prospects of Electrodes

Even though implanted electrodes significantly improve specificity and control compared to sEMG, they are not commonly adopted due to the general issues related to the FBR. Besides the abovementioned methods, multiple studies have tried to reduce the FBR by creating new biocompatible polymers. PEDOT, a newly developed conductive polymer, improves electrical conductivity while maintaining flexibility, which reduces tissue damage and, by extension, the immune response [32,45]. Another promising material is hydrogels, which provide a more biocompatible interface between electrode and tissue by mimicking tissue’s soft, hydrated environment [37]. The design of the electrode can be modified to reduce micromotion, which also reduces the FBR by using designs such as soft electrodes, which adapt to the movement of surrounding tissues to help reduce constant irritation. Minimizing the size of the electrodes can reduce the exposure of foreign material to the body, thus reducing the overall immune response [45]. This same group of researchers also introduced shape memory polymers (SMPs). A more advanced electrode design may be able to further reduce tissue irritation by allowing electrodes to be inserted rigidly and then soften once inside the body, which reduces the mechanical strain on the tissue [45]. Similarly, surface modifications such as nanostructured and micropatterned surfaces that mimic the extracellular matrix can reduce the FBR, promote cell adhesion, and reduce immune activation [32].

Another approach to improve electrodes in general is using drug-eluting coatings, which slowly release anti-inflammatory drugs such as dexamethasone to suppress the immune response, helping to reduce inflammation and scar tissue formation surrounding the electrode [41]. Coatings with bioactive molecules encourage better device integration with the surrounding tissue by promoting nerve regeneration and reducing glial scarring [32]. 

In contrast, some studies showed that low-intensity electrical stimulation may have potential in reducing the FBR. Another study reported that simulating implanted electrodes immediately after surgery could promote better integration with nerve tissue and reduce inflammation [41]. Coker et al.’s [32] C-loop systems are designed to detect the onset of an inflammatory response and respond automatically by adjusting stimulation parameters or administering targeted anti-inflammatory treatments. These adaptive systems hold significant potential for reducing FBR by minimizing inflammation and improving the compatibility of implanted devices. Currently, less invasive surgical techniques are being optimized to reduce tissue damage during electrode implantation, another contributor to scar tissue formation and inflammation, which increases electrode impedance [45].

Hybrid Electrode Designs

Another promising development is a hybrid design between a cuff and a sieve electrode. The CASE integrates micro-fabricated CASE into a single unit to benefit from both types' advantages while reducing their weaknesses [46]. Although hybrid electrode designs remain a relatively unexplored area of research, with few studies addressing their implementation, they demonstrate unique efficacy.

The sieve section of the CASE comprises multiple ring-shaped electrode sites designed to interface with individual fascicles within the nerve to provide high selectivity for both recording and stimulation. The sieve electrodes can access the fascicular topography directly, which enables precise targeting of specific nerve fiber populations. 

Combining CASE offers high selectivity (from the sieve electrodes) and enhanced stability (from the cuff electrodes). The cuff electrodes can minimize micromotion and protect the sieve electrodes from mechanical stress and crosstalk from surrounding tissues. A combination of both electrodes provides increased contact points and thus can potentially improve signal quality and reduce noise interference. Not only is the CASE better for signal acquisition, but the integrated design of the CASE can also simplify the implantation procedure compared to implanting the CASE separately by reducing the amount of foreign material that is introduced to the body. 

Although promising, the CASE design still faces many challenges. Further investigations are required to improve CASE by optimizing the size, shape, and arrangement of the CASE sites, depending on patient needs and target nerves based on amputation level. Also, the long-term stability and biocompatibility of the CASE and how it impacts nerve regeneration and function need to be evaluated through clinical trials to confirm its safety and performance before it can be widely used in patients. 

Exploring new materials and coatings can further improve biocompatibility, reduce the FBR, and increase the implant's stability in the long term. Also, wireless power and data transmission systems that can eliminate percutaneous leads to minimize infection and chronic inflammation risk can also lead to major improvements in the CASE system [46].

Regenerative Peripheral Nerve Interfaces

RPNIs are another up-and-coming prospect for sustainable PNIs. RPNIs function as a biological interface within prosthetic systems to improve control and restore sensory feedback for individuals with limb loss. They act as a bridge between the residual nerves and the prosthetic device, allowing for more intuitive and natural interaction [36,38,47,48]. 

A regenerative peripheral interface is created by suturing the distal end of the transected nerve into the muscle graft, creating an enclosed biological PNI [47]. Then, the implanted nerve requires regeneration, revascularization, and reinnervation, which typically takes around three months in humans before it can be considered an RPNI [48]. This process creates a stable, peripheral nerve bioamplifier of efferent motor action potentials that produces high-amplitude EMG signals to control a prosthetic device [38]. Multiple RPNIs can be created on a single nerve by separating individual nerve fascicles within a nerve bundle and wrapping a small piece of muscle around one or more of the fascicles. Once the fascicles are separated, a small piece of muscle is wrapped around one or more of the fascicles to create an RPNI. This technique is especially beneficial when the nerve has already sorted into predominantly motor and sensory fascicles, since it allows for potentially greater selectivity [36]. 

After this phase, electrodes can be implanted in the RPNI to create the neural interface, which can be connected to the signal processor and microcomputer via wires. Combined with OI, the medullary canal provides a stable environment for the prosthetic. Table 2 summarizes the characteristics and usage of commonly used electrodes in RPNIs. 

**Table 2:** Comparison between custom bipolar percutaneous intramuscular electromyography electrodes, bipolar wire electrodes, and indwelling bipolar EMG electrodes. EMG: electromyography; mm: millimeter; RPNI: regenerative peripheral nerve interface

Characteristics/type of electrode	Custom bipolar percutaneous intramuscular electromyography electrodes	Bipolar wire electrodes	Indwelling bipolar EMG electrodes
Contacts	2, 10 mm, and 5 mm in length, spaced 5 mm apart, with a diameter of 0.75 mm36	Smaller inter-contact distance of 1 mm [38]	Larger inter-contact spacing of 10 mm, which captures larger amplitude efferent motor action potential signals [38]
Usage	Percutaneous insertion, which can record signals and stimulation without an implanted connector [36]	Electrodes are inserted percutaneously for a single session [38]	Surgically implanted within the RPNI muscle and are usually used for long-term recordings, can record stable EMG for up to 7.5 years [38]

Electrodes are usually implanted within the muscle tissue of the RPNI instead of directly on the nerve, which dampens electrode micromotions to reduce mechanical trauma and helps maintain long-term stability of the electrode. The muscle tissue also provides insulation for the electrical signals generated by the electrodes, preventing signal leakage and ensuring that the stimulation or recording is localized to the desired area within the RPNI [38]. 

The placement of the electrode within the RPNI can influence the stimulation threshold required to elicit sensation or the quality of recorded motor signals. Since the density of sensory and motor axons is not uniform throughout the RPNI, some electrodes placed in regions with a higher concentration of relevant axons require lower stimulation thresholds and produce stronger signals [49]. To address the variability in axon density and potentially enhance signal selectivity, future systems could consider using multi-sensor arrays, which would consist of multiple electrodes distributed within the RPNI. These arrays could allow for more precise targeting of specific axon populations and possibly provide more nuanced control and feedback [50].

RPNIs generate control signals by recording EMG activity from muscle grafts that have been reinnervated by residual nerves [48]. When an individual intends to move their phantom limb, the corresponding efferent motor signals are amplified by the RPNI and recorded by implanted electrodes. These amplified EMG signals are then processed and decoded by algorithms to identify intended movements, which are translated into commands for the prosthetic limb [29]. This enables control of various grasp patterns, individual finger movements, and even more complex actions like thumb opposition, allowing for a more natural mapping of intended movements to prosthetic actions [47].

RPNIs enable sensory feedback by stimulating the afferent sensory axons within the reinnervated muscle graft. This stimulation can evoke sensations that are perceived as originating from the missing limb, providing feedback on the prosthesis's interaction with the environment. Due to technological limitations such as higher stimulation thresholds that require stronger electrical currents to elicit sensations, increasing the risk of discomfort or damage to the nerve [36], current literature primarily discusses sensations of tingling and kinesthesia. Future work could investigate the potential for restoring a wider range of sensory modalities through RPNI stimulation, including touch, pressure, temperature, and proprioception, to enhance the feeling of embodiment, improve control accuracy, and facilitate more natural and intuitive use of the prosthesis [49]. 

RPNIs have also been associated with a reduction in neuroma and phantom limb pain, possibly due to the redirection of regenerating nerve fibers into the muscle graft, preventing the formation of painful neuromas and potentially promoting more normalized sensory signaling [50]. 

RPNIs provide multiple independent control sites, allowing more precise and dexterous control of multi-articulated prosthetic hands [29,48]. This can improve the functionality of the prosthesis and reduce reliance on compensatory movements. For example, RPNIs enabled two participants to control a virtual prosthetic hand with high accuracy, including individual finger movements and different grasp patterns. In addition, a participant maintained real-time prosthetic performance above 94% accuracy for 604 days without recalibration, demonstrating that RPNIs were particularly valuable for predicting intrinsic hand movements, which are essential for precise object manipulation [29]. 

RPNIs have demonstrated long-term signal stability, with recorded EMG signal quality remaining high for up to 1054 days (approximately three years) in one participant from Vu et al. [47]. The biological nature of the interface allows natural integration with the surrounding tissues and reduces the risk of foreign body reactions or electrode migration, common in other PNIs such as cuff electrodes, as mentioned in Zbinden et al. [37]. Cuff electrodes are implanted directly around the nerve, which can trigger abnormal morphology at the nerve cuff level, nerve inflammation, and FBRs. These reactions can lead to nerve damage and mechanical trauma, ultimately affecting the quality of signal recordings. In contrast, RPNIs place the electrode within the muscle tissue rather than directly on the nerve, dampening electrode micromotions and reducing the risk of mechanical trauma [36]. The RPNI muscle tissue can maintain a consistent size and produce motor signals for up to three years when electrodes are implanted, demonstrating minimal scarring compared to other surgical approaches that often impede signal transmission and cause electrode instability [48]. Additionally, RPNIs consistently produce high SNRs, ranging from 15 to 250 across multiple recording sessions over extended periods [38], which is essential for reliable prosthetic control. 

The integration of RPNIs within ONIs may be able to surpass CASE in biocompatibility, long-term stability, and overall functionality. Since RPNIs use muscle grafts derived from the patient’s own tissue to create a natural biological interface that supports nerve regeneration, it reduces FBRs, inflammation, and scarring in contrast to CASE, which directly interacts with nerve tissue. RPNIs may also be able to offer better long-term signal stability due to the reinnervated muscle grafts, which act as natural amplifiers for neural signals. After the transected nerve regenerates into the muscle graft, the graft stabilizes and consistently produces high-amplitude EMG signals. Due to the presence of electrodes within the muscle tissue, rather than directly on the nerve, less micromotion and mechanical stress can be at the interface, further preserving signal integrity over time. Additionally, RPNIs prevent neuroma formation and reduce phantom limb pain by redirecting regenerating nerve fibers into muscle grafts, in contrast to CASE, which directly interacts with nerve tissue. Sensory feedback may also be better in RPNIs, as reinnervated muscle grafts allow localized stimulation of sensory axons, which reduces the risk of discomfort or nerve damage seen in CASE. RPNIs are also modular, since multiple RPNIs can be created by separating nerve fascicles and wrapping each with individual muscle grafts. Thus, surgeons can create multiple independent control sites, enabling even more complex prosthetic movements. Paired with a scalable ONI design, which provides a robust casing for managing the increased data flow from multiple RPNIs, RPNIs with ONI have the potential to provide a more natural, stable, and comfortable interface for prosthetic systems. 

Future investigations should integrate RPNIs with advanced prosthetic hands using sophisticated control algorithms to develop robust and reliable systems that can seamlessly translate user intent into prosthetic action while providing meaningful and intuitive sensory feedback similar to decoding models and mapping parameters in myoelectric control [51].

Challenges remain in optimizing RPNI design, placement, and long-term stability. Further research is needed to develop electrode designs that minimize tissue reactions and ensure long-term biocompatibility [47], improve techniques for precise electrode placement within the RPNI to maximize signal selectivity and minimize stimulation thresholds [38], explore the use of multi-sensor arrays to enhance control and feedback capabilities, and investigate the potential for delivering a wider range of sensory feedback modalities, including touch, pressure, temperature, and proprioception [29].

Clinical Ethics in Experimental Neural Interfaces

Clinical trials have achieved significant technological advancements in the world of PNIs, enabling bidirectional communication between the nervous system and prosthetic limbs for intuitive control and sensory feedback. Recently, there has also been a boom in AI-based decoding algorithms due to their enhanced accuracy, responsiveness, and ability to adapt to individual patients, improving embodiment rates [52,53]. As a result, many companies, such as IotaBioscience [54], Neuros Medical [55], and Neuronoff [56], are commercializing neural prosthetics through developing and testing experimental interfaces in clinical trials.

However, there are many limitations to these trials, such as a limited understanding of the long-term mechanisms involved, limited patient selection, trial design limitations, and ethical regulation gaps. Bidirectional prosthesis requires an understanding of how peripheral nerves and the device interface with each other, but there are limited long-term studies on post-interfacing tissue and recovery. Thus, it is difficult to predict how the interface will change over time and to what extent it will cause adverse reactions such as scarring, immune responses, or electrode degradation, affecting both the patient and the long-term performance of the prosthesis [57]. Additionally, due to the adaptive nature of bidirectional prosthesis, where real-time calibration is needed, traditional safety and efficacy evaluations currently used in pharmacological research do not fully apply [58]. Moreover, due to the small number of patients that enroll in these trials, the results of long-term studies are use-case-specific and often not generalizable [59]. The adoption of AI also involves unclear legal liabilities and privacy violation regulations around device failures and decision-making errors by AI systems [60]. 

A critical but often overlooked issue in implantable neural interfaces, such as osseointegrated prosthetics with PNIs, is the logistical and ethical challenge posed by device failure or corporate abandonment. As neurotechnology advances, concerns grow regarding long-term patient safety, maintenance, and accountability when companies cease support or go bankrupt.

Thus, there are many ethical considerations surrounding PNIs. First, due to the complex nature of the prosthesis, patients may not fully understand the long-term consequences of implantation, the irreversibility of certain procedures, and the risk of device obsolescence. Additionally, there are risk-benefit considerations, including infection, electrode migration, and scar tissue formation, among others, but there are significant benefits, such as restoring lost functions, mobility independence, and reducing chronic pain, which enhances overall well-being [47]. Ethical trials need to make sure that potential benefits significantly outweigh the risks involved. Furthermore, there are many privacy and security risks as they continuously collect data, such as unauthorized data extraction and unsecured data transmission [61]. AI-driven datasets may also contain biases as they are trained on limited datasets, leading to unequal effectiveness across demographics [62]. There is also the issue of a company ceasing support for a prosthesis, as patients may rely on the device for daily functioning. Companies should be legally required to provide long-term support plans, fund device maintenance and updates, and establish trust funds for explantation if support ceases completely [63].

To ensure ethical and responsible clinical trials, comprehensive psychological assessments that go beyond evaluating physical function to include cognitive and emotional well-being, using metrics such as user acceptance, identity perception, and cognitive integration, have been proposed. These subjective quantitative metrics can improve patient experience and embodiment in addition to standard metrics in current assessments [63]. Secondly, stronger data security and privacy measures are essential, including the implementation of Neural Data Protection Impact Assessments and ensuring neural data encryption and secure transmission protocols [58]. Thirdly, AI bias auditing and explainability must be prioritized to ensure that algorithmic decisions are interpretable to prevent unintended neural misalignment by inclusively representing diverse demographics in the training data [64]. Finally, clear liability and long-term support requirements should be established, holding companies legally responsible for device maintenance and mandating post-trial obligations, such as long-term patient support and insurance policies for explantation [65].

## Conclusions

The integration of ONIs with PNIs enhances prosthetic control by enabling precise signal decoding, motor command generation, and sensory feedback, improving dexterous movement and user embodiment. However, their invasive nature presents challenges, including surgical risks, inflammation, and long-term signal degradation, necessitating further research. Regenerative RPNIs offer a promising alternative by utilizing muscle grafts to enhance biocompatibility, reduce neuroma formation, and provide stable, high-amplitude signals. Advances in materials science and machine learning-driven design further support the development of durable, patient-specific neural interfaces. Ultimately, the combination of osseointegration, PNIs, and RPNIs represents a transformative approach to prosthetic control, with the potential to restore independence and significantly improve the quality of life for amputees.
